# Combatting Homelessness in Canada: Applying Lessons Learned from Six Tiny Villages to the Edmonton Bridge Healing Program

**DOI:** 10.3390/ijerph17176279

**Published:** 2020-08-28

**Authors:** Anson Wong, Jerry Chen, Renée Dicipulo, Danielle Weiss, David A. Sleet, Louis Hugo Francescutti

**Affiliations:** 1 School of Public Health, University of Alberta, Edmonton, AB T6G 1C9, Canada; aawong@ualberta.ca (A.W.); jerry5@ualberta.ca (J.C.); dicipulo@ualberta.ca (R.D.); 2 Northern Alberta Institute of Technology, Edmonton, AB T5G 2R1, Canada; d-weiss@live.ca; 3 The Bizzell Group & School of Public Health, San Diego State University, San Diego, CA 92182, USA; davidasleet@gmail.com; 4 School of Public Health, University of Alberta, Edmonton, AB T6G 2E9, Canada

**Keywords:** homeless(ness), Tiny Villages, Bridge Healing Program, homeless patients, emergency housing

## Abstract

Emerging evidence shows that homelessness continues to be a chronic public health problem throughout Canada. The Bridge Healing Program has been proposed in Edmonton, Alberta, as a novel approach to combat homelessness by using hospital emergency departments (ED) as a gateway to temporary housing. Building on the ideas of Tiny Villages, the Bridge Healing Program provides residents with immediate temporary housing before transitioning them to permanent homes. This paper aims to understand effective strategies that underlie the Tiny Villages concept by analyzing six case studies and applying the lessons learned to improving the Bridge Healing Program. After looking at six Tiny Villages, we identified four common elements of many successful Tiny Villages. These include a strong community, public support, funding with few restrictions, and affordable housing options post-graduation. The Bridge Healing Program emphasizes such key elements by having a strong team, numerous services, and connections to permanent housing. Furthermore, the Bridge Healing Program is unique in its ability to reduce repeat ED visits, lengths of stay in the ED, and healthcare costs. Overall, the Bridge Healing Program exhibits many traits associated with successful Tiny Villages and has the potential to address a gap in our current healthcare system.

## 1. Introduction

A report from 2016 estimated that 235,000 Canadians experience homelessness every year, indicating that homelessness continues to be an ongoing problem in Canada [1]. To address this, Tiny Villages have emerged as an affordable and popular response in several countries, including Canada, the United States, and parts of Europe [2,3].

The philosophical underpinning of Tiny Villages for the homeless originated from the Tiny Village social movement that emphasizes a shift toward more equitable and affordable housing by dramatically downsizing living spaces to around 400 sq. ft., in [4].

Homelessness has been rising in Edmonton due to factors, such as a decrease in affordable housing, job losses, rising eviction rates, and overcrowded shelters. Many people sleeping in shelters are required to vacate the shelters each morning, leaving them without a place to go. As other research has found, assessing the relative effects of housing, economic, safety net, demographic, and climate factors on homelessness rates helps support the availability and affordability of housing for the homeless, two factors that are “… often a community’s best line of defense against homelessness” [5].

In Edmonton, Canada, the Bridge Healing Program hopes to use emergency departments (EDs) as a gateway to temporary housing for the homeless. The pioneer of the project, Dr. Louis Hugo Francescutti, is an emergency physician at the Royal Alexandra Hospital. After witnessing recurring homeless patients in the ED and the lack of hospital support post-discharge, Dr. Francescutti sought to change this. The proposed plan of the Bridge Healing Program is to build units, similar to Tiny Villages, near the ED, that can house patients who are experiencing homelessness. Patients will stay for a maximum of 28 days before permanent housing is found for them. Once residents are moved into permanent homes, the units will then be made available to other patients experiencing homelessness.

This paper has four aims. First, we will briefly define Tiny Villages and their philosophy. Second, we will examine six case studies across the world to determine what the common successful strategies employed by Tiny Villages are and to identify some of the common problems they face. Using our findings, we will suggest an ideal model of a Tiny Village to improve the chances of success in a community. Finally, using our model, we will apply it to the Bridge Healing Program to determine whether implementation in the context of the Bridge Healing Program would prove effective.

## 2. What are Tiny Villages, Criteria for Case Inclusion, and Methods

Tiny Villages, synonymous with Tiny House Village, Tiny Houses, Tiny Homes Village, Homeless Villages, Social Bite Villages, Dignity Villages, and NestHouses etc., all refer to 80 to 700 square feet of housing space where people experiencing homelessness can live for extended periods before transitioning to permanent housing [6]. Compared to homeless shelters, Tiny Villages have been described as being more cost efficient, faster to implement, and are known for providing wrap-around services in the form of health care, social services, financial counselling, childcare, and mental health and addiction counselling [6,7]. Tiny Villages are generally operated with fewer restrictions than shelters and are often self-run with minimal supervision [8]. When Tiny Village residents in Seattle were interviewed in 2017, they described the village as being self-empowering and safer than alternative options. Tiny Villages remain a viable, yet flexible, model that can address homeless housing needs and adapt to different environments and local policies.

The six case studies that we looked at included Dignity Village, Occupy Madison, Homes for Heroes, Social Bite Village, Kenton Women’s Village, and the Dwellings. When deciding which projects to include, we prioritized Tiny Villages (as opposed to other affordable housing options) as they were deemed most similar to the Bridge Healing Program—mainly through the inclusion of wrap-around services, their intended size, and their goals. For instance, the Bridge Healing Program aims to run the program with less supervision than traditional affordable housing projects and will also help residents find permanent homes. Of the Tiny Villages we studied, we included projects with variable sources of funding (independent and government funded), stay times, services, and target populations (see Table 1 for a summary of these differences). By using these six different projects as case studies, we were able to compare and contrast their plans to identify common strategies that proved most effective and also common challenges. We used qualitative methods which included recorded interviews with residents and staff, newspaper articles detailing their development, and studies that followed residents post-graduation.

To gather more information about the Edmonton Bridge Healing Program, we similarly contacted and interviewed students from the Northern Alberta Institute of Technology (NAIT), who are helping with planning and development, and also obtained the initial project plan. This gave us insight into their goals and methodology, allowing us to evaluate their program relative to the six case studies.

## 3. Case Studies

### 3.1. Dignity Village (Oregon, United States)

Dignity Village is a community-organized Tiny Village that houses 60 people every night [10]. Starting as a tent city in the 2000s, Dignity Village has since become a registered non-profit and is governed by a democratically elected board [10,11,12]. The village emphasizes this democratically styled self-governance fosters self-empowerment and community control as a unique strategy to combat homelessness.

A Village Intake Committee selects memberships with limited entry barriers and even accepts clients with a criminal history [13]. Members are required to abide by community bylaws, including a maximum stay of two years, no alcohol or drugs on premise, no violence or theft, and a minimum 10 h “sweat equity” volunteer work commitment per week [13]. Membership also requires a USD $35 fee per month [12].

Over the years, houses have been built by volunteers, transitioning from tents to more permanent wooden tiny houses [12]. Utility bills such as water, electricity, garbage collection, and the internet are paid for by the residents. There is no plumbing in the houses, so portable toilets and propane heaters are made available [14].

Dignity Village is unique in both its origins and setup. Dignity Village provides “transitional housing” but lacks on-site case management treatment and social services [14]. A 2010 Oregon City study on Dignity Village reported significant cost savings compared to alternative programs [14]. In total, the village has an operating budget of USD $29,000 per year to house around 60 members and costs $4.82 to house each individual per night [14]. By comparison, sheltering costs $12.59 per night at warming centers, and $66.56 at traditional transitional houses [14]. At the same time, Dignity Village saw lower dispatches of the Portland Police (0.15 per capita) compared to the city (0.31 per capita) [14]. However, despite these successes, Dignity Village residents are evicted more often compared with other emergency shelters and transitional housing programs, which can be attributed to either its lower screening criteria, or the village’s strict self-enforcement at work [14].

At the same time, challenges involving the lack of proper sanitation, affordable housing options upon leaving, and on-site services remain prevalent [14,15]. Due to a lack of funding, the program cannot afford private showers and bathrooms [15]. Residents have cited instances of overflowing feces and urine in the bathrooms [15]. Additionally, one propane-heated shower is meant to provide bathing to all 60 residents. If Dignity Village were to install a shower and toilet system, it is estimated to cost an initial $15,000 (USD) and $900 for monthly maintenance [14]. These renovations could dramatically improve sanitation but would simultaneously increase the costs to fund the project.

Another primary issue of the program is the lack of affordable housing post-graduation. With a minimum wage of $9.25 and average monthly rent costing $1,160 for post-graduation housing in the community, it is unlikely that residents will be able to afford housing after they leave the program [15]. As a result, about 70% of residents leaving Dignity Village end up returning to homelessness [14]. Residents conveyed dismay with the two-year maximal stay provision, stating that they required more time to save enough money to afford housing post-graduation [15].

Finally, the lack of professional staff makes it difficult to provide services on-site. These issues are further compounded by the geographical isolation from the city, which is a 30 to 45 min bus ride from downtown Portland [16]. More recently, Dignity Village has partnered with local organization “JOIN” to provide on-site social services that help with referrals to “employment support, social security applications, housing searches, healthcare access, mental health and addiction support, obtaining ID and other documents, and more” [17].

### 3.2. Occupy Madison (Wisconsin, United States)

Occupy Madison is a unique approach to Tiny Villages, where instead of rent, residents exchange volunteer hours for shelter [15]. The village consists of five tiny houses and provides shelter to chronically homeless people [15]. Each house is 98 square feet and contains working electricity but lacks running water [18]. Instead, communal showers and toilets are located in a central building accessible to all residents [18]. Residents are required to volunteer 32 h of initial service to move into the program and, once admitted, must volunteer 10 h a week thereafter [18,19]. Volunteer activities include maintaining the shelter, cleaning bathrooms, working the garden, and making goods that can later be sold [15]. Unique to the Occupy Madison project is that there is no time limit on the residents’ stay. The project allows residents to stay for as long as necessary until they are ready to live independently [15].

Occupy Madison raises a small amount of money by selling goods made in the on-site workshop and plants grown in the on-site garden [20]. These sales have been reported as becoming increasingly successful over the past years such that in 2016, they earned a $15,000 grant from Nature. The vast majority of funds are received from charity donations, such as Bubbles Laundromat and James Reeb Unitarian Universalist church, and other private donations [15]. Occupy Madison has not sought funding from government loans/agencies as they view these funds as limiting to their freedom of operation [15]. By 2015, two years after opening, the village had only housed seven people, one of whom was evicted for misconduct and for not adhering to community guidelines [19,20].

One of the initial challenges to the success of the village was public opposition. During its development in 2014, 70% of the people owning/renting property near the village signed a petition opposing its construction [19]. Opponents argued that the village would lower property values and the influx of homeless people would increase the number of disturbances, which would also increase police presence [19]. After a year of operation, many of these fears dissipated. The Madison Police Department reported no phone calls regarding the village within one year of opening, and the surrounding property values have continued to rise [19]. Many previous opponents later retracted their opposition, and some were even reported to having volunteered in the village’s gardens [21].

The governance and sense of community have been some of the village’s strongest attributes. The board of directors includes both residents and non-residents. This shared governance contributes to residents’ feeling of self-dignity and trust between residents and the owners. Despite some of these advantages, Occupy Madison still lacks many of the services offered by other programs such as substance abuse screening and referral [18,19].

### 3.3. Homes for Heroes (Alberta, Canada)

According to the 2018 report, “Everyone counts” by Employment and Social Development Canada, approximately 4.4% of homeless individuals in Canada are veterans [22]. In Alberta, 7% of surveyed homeless individuals self-identified as having served in the Canadian military or RCMP [23]. The large number of homeless veterans is often attributed to the difficulties for some military members to successfully transition back to civilian life [24]. Approximately 11% of veterans suffer from Post-Traumatic Stress Disorder (PTSD), and many experiencing PTSD resort to alcohol or drugs to cope with their mental health, both of which are factors that contribute to homelessness.

In response to the growing number of homeless veterans, Homes for Heroes Foundation was set up to build affordable housing villages to help military veterans reintegrate to civilian life [25]. The first project was completed in Calgary in 2019, consisting of 20 tiny homes less than 300 square feet. Each home is fully equipped with a kitchen, a bathroom, a Murphy bed, basic cable, Wi-Fi, and a telephone line. They cost roughly CAD $70,000 to build and were funded in part by a CAD 1.5-million-dollar donation by ATCO, a Canadian utilities company [24].

The village partners with numerous social agencies such as The Mustard Seed, and the Department of Veterans Affairs to provide support services including a full-time support counsellor, a community garden, and a central resource office as well as peer-to-peer support programs [25]. Each individual in the village is assigned a case worker and has a personally tailored “individual support plan” to provide a structured transition to self-sufficiency [25]. The plan includes support services such as financial, mental, addictions management, family reintegration, and identifying employment opportunities. Residency is transitional (up to two years) with the ultimate goal of creating self-sufficient individuals for reintegration into society [25].

Although it is too early to comment on the program’s success, Homes for Heroes is still the first of its kind in Canada. If successful, it can serve as an important model for future tiny homes/village concepts, including its next project in Edmonton (ATCO Evansdale Village), which is expected to be completed in late 2020 or in early 2021 [26].

### 3.4. Social Bite Village (Edinburgh, Scotland)

Located in Edinburgh, Scotland, Social Bite Village opened in May 2018, with the mission of providing safe accommodations for those experiencing homelessness who are otherwise relying on hostels or bed and breakfast (B&B) lodging [27].

The main source of funding to the village is Social Bite Fund, a Scottish registered charity which seeks to combat homelessness through novel solutions [28]. The fund is supported by major fundraisers as well as its namesake business, “Social Bite”, a sandwich shop located across five locations in Scotland. Social Bite operates as a “social business model”, giving all profits to good causes and hiring a significant portion of their staff from homeless situations [29].

The village consists of 10 two-bedroom tiny homes termed “NestHouses”, alongside a community hub that accommodates approximately 20 homeless individuals overall [29]. The “NestHouses” are uniquely developed, highly insulated, energy-efficient, and made from high-quality materials [29].

The village relies heavily on non-government funding. Each house is branded and supported by £30k worth of corporate sponsorship [30]. Moreover, the village land is on loan for free from Edinburgh City Council and over 100 companies that contributed pro bono to the project. In addition, celebrities such as Leonardo DiCaprio and George Clooney have participated in Social Bite’s fundraising campaigns [30].

Cyrenians, a homeless-serving charity organization, contributes volunteers and staff to run the village [31]. The village specifically targets those currently living in temporary/emergency accommodations such as B&Bs and hostels, and the entry criteria excludes those with mental health and addiction issues [31]. Residents are expected to stay for 12 to 18 months [29]. During this time, residents have access to extensive social support including budgeting, counselling, cooking lessons, employment opportunities, and meditation facilities [29]. The goal is for the residents to obtain permanent accommodations. Significant aftercare is provided once residents move into more permanent housing [32].

Although the village only opened in May 2018, according to the 2018–2019 Social Bite Impact Report, the village has already achieved some success [32]. Of the 18 admitted individuals, five had already found employment, while another five had enrolled in courses at nearby universities and colleges [32]. While it is too early to say how successful the program is compared to other social programs, Social Bite’s unique funding structure has set it apart from other transitional housing models, and its high-quality houses have been noted as one of Scotland’s most attractive places [33].

### 3.5. Kenton Women’s Village (KWV) (Oregon, United States)

Started in 2017 as a limited duration pilot project for homeless females, the Kenton Women’s Village (KWV) (Portland, OR, USA) has since expanded and relocated in 2019–2020 to a permanent village that houses 23 women [34].

The village consists of non-gridded 4′ × 8′ micro-housing sleeping pods that provide privacy and security for each member to sleep in [35]. Each pod has no water, electricity, or heating, but is small enough that personal body heat is sufficient, thereby reducing the cost of each unit [35]. The village consists of a common building(s) with full utilities and kitchen installed in customized shipping containers, and water and garbage services [34]. In addition, the village includes a community garden for increased interaction and community building [34].

The village is run by Catholic Charities (CC) of Oregon who provide numerous supportive cares, including “case management, employment assistance, access to legal and financial services, mental and physical health care”, and a personalized plan for permanent housing transition for all women in the village [34]. Such a program is mandatory for all villagers as each resident must be actively pursuing employment or permanent housing [36]. In a 2019 survey of 9 of the 12 residents in the village at the time, the average age was 48 years, they spend an average of 5.38 years being homeless, with a range 0.67 to 14 homeless years [37]. Educational background ranged from master’s completion to no high school education [37]. As such, the village serves a diverse range of female residents.

The KWV is seen as a success as it has extended from a pilot project to a permanent housing structure. In the same 2019 survey, all villagers surveyed were satisfied with the village [37]. When interviewed about the facilities and the village, residents were largely positive, indicating the benefits of “being able to … have my own existence” and being “blown away” by the positive reception from other residents and the local community. Based on 2018 data of 24 former and current KWV residents, 14 women have already moved from the village into permanent housing with the help of Catholic Charities case managers [38]. Based on the Catholic Charities website in 2020, Kenton village has assisted 23 women into permanent supportive housing and helped them in attaining birth certificates, IDs, mental and other health services, and income development—all factors that prevent reoccurrences of homelessness [34]. Due to its overwhelming success, the Kenton neighborhood has approved a grant for KWV to expand, tripling its capacity [39].

While Catholic Charities operates the administrative and fiscal side of the village, the village is mainly self-governing and is comprised of residents, staff, and various stakeholders [38]. The village is unique in its strong local engagement as both the city and Joint Office of Homeless Service support the program [34,38]. It was reported that the village costs the city only $350,000 [36]. Contributing to such a low cost was the use of city-owned land in an industrial area unsuited for development. In addition, numerous local contractors and construction firms donated to the construction of the pod, while Portland State University School of Architecture students and an architecture firm donated time towards the construction of the building [34].

In helping gain community acceptance to tiny houses for the homeless and with its unique funding model and apparent success rate, KWV serves as a model project that could inspire future housing projects [40].

### 3.6. The Dwellings (Tallahassee, USA)

A unique approach to combating homelessness, the Dwellings applies a transitional housing philosophy to Tiny Villages. The program has three intermediate steps to help residents of various needs achieve permanent housing [41]. “The Dwellings was the first tiny home community in the area and grew out of a church’s effort to provide shelter in Tallahassee in the 1980s. By 1991, the shelter was incorporated and registered as a non-profit organization [41].”

Jackson et al., describe the project this way:

*“As the Shelter was unable to provide support services during the day to people experiencing homelessness, a group of community partners, including business owners, social service agencies, and concerned citizens, came together to fill this gap through the Renaissance Community Center (RCC). The RCC provided a place for caseworkers from various agencies to meet with clients in a central location. It was also a place for people to take showers, wash laundry, make phone calls, and use the internet. The Shelter and the RCC worked together to serve the same population and thus shared many clients. Due to the high volume of clients, both locations outgrew their spaces, leading to an agreement to locate their services in a combined building. This combined building, the Kearney Center, opened in April of 2015. In 2016, the two organizations merged into a single organization, CESC, Inc., the legal entity of the Kearney Center. Since the merger, the CESC has expanded to operate three housing developments: The Kearney Center, Westgate, and The Dwellings.*

*The Kearney Center includes free shelter for the homeless, an onsite clinic, and support services such as General Educational Development (GED) classes, prayer groups, and workforce training. The Kearney Center is considered the “first step in helping stabilize someone who is experiencing homelessness” in the three-rung ladder that CESC operates.*

*Westgate is a low-cost housing solution for those either recently experiencing or on the verge of experiencing homelessness. A variety of housing options are offered in order to ease the burden on the individual. This includes a bedroom that is often shared, communal bathrooms, and access to all of the services the Kearney Center provides to clients. Westgate is considered transitional housing and, thus, the second placement step in overcoming homelessness.*

*The Dwellings opened in December 2017 and offers housing for low to moderate income residents in addition to support services. Collectively, the physical locations of the CESC’s three housing options are situated in a triangle, providing convenient access to each other. While the Dwellings currently lacks a bus stop near its entrance, there is a circulating shuttle that will take residents between the three sites. Once at the Kearney Center, residents have access to a bus stop and all members are granted a bus pass. There are currently 89 homes in the community with about 10 homes being developed every 45–60 days. By the end of 2019, there is projected to be 130 tiny homes in three floor plans that range from 220 square feet for $600 a month to 410 square feet for $900 a month. At present, the community center and supporting services have recently been completed, and the entire project buildout will include 260 units [41].”*

## 4. Discussion and Ideal Model

The case studies that we reviewed reveal Tiny Villages as having widespread potential with many lessons learned, including:

- Governance/community
- Not in My Back-Yard (NIMBYism) and Public Support
- Funding
- Affordable Housing Post-Graduation

### 4.1. Governance/Community

One of the most promising aspects of Tiny Villages appears to be in their governance. A defining feature of both Dignity Village and Occupy Madison is the ability for residents to participate in running the program [10,11,14,19]. This approach is important as low self-esteem is often associated with homelessness and can lead to other negative consequences like depression and poor health [42]. By giving residents responsibilities, the program shows that it has faith in its residents to make meaningful decisions, which can translate into self-empowerment [15]. Simultaneously, this system of governance also fosters equity. Running a Tiny Village requires a diverse team of people, many from vastly different socioeconomic backgrounds [15]. As such, a hierarchy can often be established between residents and non-residents. Opening up governance to all people can help break down this power dynamic [15].

Simultaneously, a limitation with residents participating in governance is that they are only allowed to govern for a limited time. As residents graduate from the program, Tiny Villages will constantly need to look for and train replacements. This issue has been previously identified as a concern with the Occupy Madison governance system [15]. Instead, it may be more beneficial to have a permanent core team that can contribute continuity to the program in addition to recruiting residents.

Ultimately, our tiny village case studies reveal the importance of involving residents in governance to create a stronger community. Future Tiny Villages could implement this as a way to break down the barrier between staff and residents.

### 4.2. NIMBYism and Public Support

NIMBY, standing for “Not In My Backyard,” is a common phenomenon whereby residents oppose development as unsuitable in their area for a variety of reasons [43]. Such opposition can hamper project design and completion, increase costs, and/or reduce site availability.

Of the six case studies, many have experienced public and sometimes government pushback. For example, in Occupy Madison, the project faced strong community opposition with petitions expressing fears of “lower property values, endanger safety and pose sanitation problems” [19]. Even the local police department opposed the project, fearing the need for increased police service. In the Dwellings, residents filed a lawsuit opposing the project for fear that such a project would be a “dumping ground for ex-cons and the homeless” [44]. However, despite such opposition, Madison city councilors unanimously approved the project in part due to the city’s Plan Commission recommendation [45,46,47]. This suggests that governmental committees, such as Edmonton’s Urban Planning Committee, may play key roles in helping move such projects forward despite NIMBY opposition. At the same time, in both Occupy Madison and the Dwellings, public opinion improved once the project was complete and the NIMBY fears had not materialized [19,41]. On the other hand, Dignity Village was unique in that its founding was only city sanctioned after years of campaigning and after the courts overturned the city’s camping ban law. Such law touched on the issue of the “criminalization of homelessness”, and highlights the need for city co-operation for successful projects [48].

Interestingly, Social Bite Village is unique in that we could not find public opposition to the project. Co-founder Josh Littlejohn stated that he has not come up “against local opposition to the village” and suggested that its location outside any residential streets was the reason [49]. However, such lack of opposition could also stem from the organization’s deep community roots, having had successfully operated a homeless/volunteer run cafe and restaurants in the communities. Moreover, Social Bite has had celebrity endorsements, including Leonardo DiCaprio, which may help public acceptance of such projects [30].

Additionally, the Homes for Heroes (HOH) expansion into Edmonton’s Evansdale community has faced community opposition. An open letter by the community league expressed concerns for the project for taking up greenspace, potentially increasing traffic volumes, and that there are alternative sites in other communities that may be more suitable [50,51,52]. Additionally, Dave Howard, HOH president, stated that the Calgary HOH project has faced “concerns about veterans endangering the neighbourhood children” [53]. These evidences suggest that future tiny home projects in Edmonton and Alberta could also face NIMBYism.

Nevertheless, there are ways to combat this initial opposition. For instance, Occupy Madison relies heavily on media [15]. They have shared photos on social websites like Buzzfeed and Facebook, and there have also been many newspaper articles written about their goals and development [15]. Media coverage can garner public attention, which, in addition to helping raise donations, can help dispel many negative stereotypes. Some villages used public endorsements by celebrities to gain acceptance [30]. Above all, though, it appears that one of the best methods of addressing opposition is to publicize successes, proving that the opposition is misplaced. Within one year of opening, Occupy Madison saw opinions dramatically transform with many attributing this to smooth implementation, lack of violence, and increased property values by the year’s end [19].

### 4.3. Funding

As the main goal of these Tiny Villages is to provide short-term equitable and affordable housing rather than making a profit, raising the necessary finances to support the homes can be challenging. For example, The Dwellings project in Tallahassee struggled with funding constraints and this limited their development [41]. Methods of raising funds include government grants, loans, charity donations, and rents. Government funding can sometimes provide large amounts of funding otherwise unavailable in the community, allowing greater services and more staff. Simultaneously though, government funding often has restrictions on how the funds can be spent, limiting creative implementation. For instance, in 2018, the provincial government of Alberta, Canada, provided funding to build affordable housing through their Housing Development and Renewal Capital Program [54]. Despite this, funding was restricted to certain applicants, the program had to align with the government strategic plan, and the rent was expected to be 30% of the tenants’ incomes. To some, expectations like these may make working with the government unfavorable. For instance, Dignity Village and Occupy Madison have shown an aversion to government subsidies and continue to seek funding through private investors and local charities instead [15]. Consequently, because of their lack of funding, these projects lack many services [16,19].

This observation does not imply, however, that large sums of funding from non-government sources cannot be found. Instead, these funding sources may require more time and creativity to identify and obtain. KWV and Social Bite Village are two examples of Tiny Villages that offer services like personalized plans, mental and physical health care, cooking lessons, and counselling funded primarily from non-government sources [29,33]. KWV lowered its costs because contractors and students volunteered their time constructing the building [34]. Similarly, Social Bite Village relies on corporate sponsorships, volunteers from the homeless charity, Cyrenians, and their Social Bite sandwich shops to support their efforts [29,30,31]. As such, if government sources are not sought, costs can be reduced by decreasing services offered or relying on volunteers and seeking donations. In an ideal model, one would be able to find a source of revenue that has few restrictions but could also sufficiently cover all expenses.

### 4.4. Affordable Housing

While Tiny Villages are very important, their use provides little help in combating homelessness unless affordable housing is available upon program graduation. In a city where there are affordable housing options, the main goal of these Tiny Villages should be to connect homeless people to these homes. During their stay in the tiny village program, staff can assist residents in finding a job or financial support (e.g., low-income government support, financial aid for those with disabilities, etc.). Additionally, staff can help with other challenges such as trauma, addiction, and mental illness that could hinder residents from living independently.

If the program is in a city where affordable housing is not a viable option, this becomes increasingly problematic, as shown by 70% of postgraduates from Dignity Village returning to homelessness after program graduation [14]. A potential solution may be to make the Tiny Villages permanent housing where people can move in indefinitely, similar to Occupy Madison [15]. In this model, residents live independently as if they were renting an apartment or house. They would only be evicted if they failed to pay rent or did not follow program guidelines. In this way, rather than finding affordable houses, the Tiny Villages transition into affordable houses. The goal shifts from helping people graduate to maintaining the village for its residents.

## 5. Ideal Model

**Figure 1:**
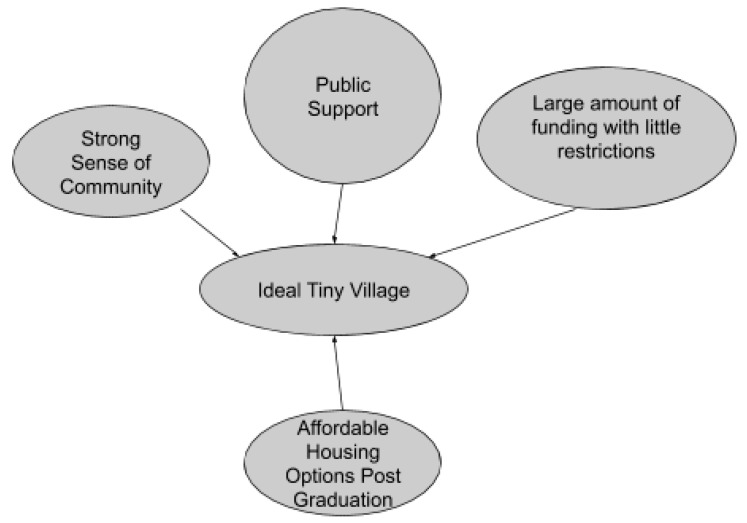
Key elements of an ideal tiny village.

As such, based on the six case studies, it appears that the best tiny village should have a strong sense of community and public support. If the tiny village is facing opposition, there should be attempts to gain support through publicizing success through media coverage (including social media) and anticipating opposition and combatting those with facts. Sources of funding should contain few restrictions but cover the costs of all required services. Finally, there should be affordable housing options after graduation. These key elements are integral in creating an ideal tiny village (Figure 1).

## 6. Bridge Healing and Applying Lessons

The proposed model of the Bridge Healing Program seeks to address a unique gap that exists between healthcare and those experiencing homelessness. The ED of the Royal Alexandra Hospital (RAH) is an inner-center hospital in Edmonton, Alberta [55]. Due to the inner-city location, the RAH ED sees a significant number of patients experiencing homelessness. The Bridge Healing Program can help redirect these patients from the frequent visits to the RAH ED by providing immediate short-term housing before transitioning them to a long-term, permanent housing solution. Using the housing first concept, the project hopes to use the RAH ED as an access point for redirecting individuals to Bridge Healing and Tiny Villages.

The proposed path is for nurses to interview patients during the triage and registration process and ask them if they have a place to sleep that night. Patients will then be seen and treated by the physician. Depending on the severity of the medical issue, patients will either be admitted or discharged. If the patient is to be discharged, the social work department will be contacted for support and discharge planning and assessment. If the patient is deemed an appropriate candidate, they will be referred to Bridge Healing. If the patient is admitted to the hospital, the medical issue will be treated, and then upon discharge, the same discharge process will be followed. Patients may not be deemed appropriate candidates for Bridge Healing if they are unable to live independently or have chronic health conditions that require medical intervention. It is anticipated that patients will stay for a maximum of 28 days before being placed into permanent housing. Patients will have a reasonable plan for transition to permanent housing that is detailed in their referral assessment. The proposed project includes one building with 12 independent units within close proximity to an inner-city ED.

During the resident’s stay, Jasper Place Wellness Centre (JPWC) will provide wrap-around services such as medical services, mental health services, cooking classes, education, and employment/skills training [56]. JPWC has four additional, for-profit, social enterprise businesses: JUNK4GOOD, SIMITU, Evergreen Recycling, and SALVAGE [57]. These companies employ over 40 vulnerable, low-skilled Edmonton residents and have agreed to provide potential occupation to graduates [57]. It is estimated that a 12-unit building will cost $700,000 to run every year for the next 5 years.

This project model has been developed by several Northern Alberta Institute of Technology (NAIT) capstone teams. As of April 2020, there have been four capstone teams from various disciplines working on the design and concept of this project model. Two bachelor of technology management (B.Tech), one bachelor of business administration (BBA), and one digital marketing information technology (DMIT) capstone teams have begun the foundational work for this project. Moreover, many other hours have been volunteered by key stakeholders, graphic designers, and community members. At the time of publication, the project has developed marketing collateral to address the potential issue of cost (funding). A phone app has also been developed for communication between RAH and Bridge Healing and the project management aspects.

### 6.1. Services and Team

One of the most promising aspects of the Bridge Healing Program is the services offered, specifically the medical and mental health services. Looking at Occupy Madison, we found that a lack of social services leaves those suffering from addiction and mental illnesses particularly jeopardized [18]. Given that the target demographic of this project is patients in the ED, where one in 10 Canadian patients suffer from addiction or mental illness, these services will likely play a vital role in determining its success [58].

Additionally, the Bridge Healing Program has assembled a team of volunteers and local charities such as the NAIT capstone team and JPWC. This community aspect mirrors KWV which, by working with local contractors and the Portland State University School of Architecture, was able to mitigate the cost of the project [34]. Improving on such ideas has involved volunteers and local charities not only in architectural and building services, but also in graphic design and social relations. One especially unique aspect of the project is the development of a phone app which will facilitate easier communication between team members. Of our six case studies, none have attempted to implement something like this; to our knowledge, no Tiny Village or affordable housing projects currently have such a system in place.

### 6.2. Costs

One of the major challenges of this project is its high costs. With operating costs of CAD $700,000, for the next five years, this makes Bridge Healing more expensive than all the case studies illustrated here. KWV is the most expensive village that we studied, and it only requires USD $350,000 to run [36]. Furthermore, despite largely depending on non-government funding, KWV did use city-owned land as a means to diminish their costs. As such, unless the Bridge Healing Program has affluent and generous donors, they will likely need to seek government funding. Grants could be sought, for example, from the Alberta Housing Development and Renewal Capital Program as well as other local and provincial funders [59].

To further opportunities for financial support, following the pilot project, data will be collected and analyzed and presented to health organizations, demonstrating the business case for the model.

### 6.3. Time Limit

Based on our case studies, we find that each project has allowed residents to stay for a minimum of 18 months, with some allowing residents to stay indefinitely. Dignity Village alone has a two-year maximum stay, but internal reports have indicated that residents are dissatisfied with this period. In fact, residents often overstay, with average stays ranging from 24–36 months—the major reason cited being the inability of residents to find affordable housing in time [60]. By comparison, Bridge Healing anticipates that its residents will stay for a maximum of 28 days. This shortened time frame could be worrisome, unless the staff can actively work to find residents permanent housing. Furthermore, due to the short stay time, it is unlikely that residents can participate in governance in any meaningful way. The project could explore giving leadership opportunities to graduates, which can provide important feedback to better understand how the project can improve from a resident perspective.

### 6.4. Gaining Public Support

Given that there is extensive evidence of NIMBYism for tiny home projects around the world and even in Alberta, measures tackling NIMBYism have to be addressed for any future projects. In a Canadian literature review on NIMBY by Alberta Centre for Child, Family and Community Research, the review establishes frameworks to address NIMBYism for affordable housing. Their conclusions include the fact that [61]:

- “Numerous studies have failed to find a relationship between [affordable] housing developments and increases in neighbourhood crime”, and that inviting police and emphasizing security should be a priority in public meetings.
- No link between affordable housing and decreased property values have been found.
- Arguments that affordable housing will strain infrastructure (i.e., such as increasing traffic and parking, as highlighted by HOH community letter), can be valid or a mask for discriminatory opposition. Such concerns can be addressed by the fact that the project must meet community planning and engineering requirements, and higher density in housing overall requires less infrastructure building by the city.
- Education and public engagement is key to reducing stigma around affordable housing, including effective, tailored, and robust communication platforms such as fact sheets, website, newsletters, presentations, etc.
- Project planning should include “developer, people and agencies that would benefit from the project” from an early stage, and selection of the neighborhood for the project should be carefully considered including potential surveys about public reaction to such a project.
- Effective monitoring strategies should be set up to gauge the success of public engagement and to establish the long-term value of projects to the neighborhood, with the potential to sway public opinions post-project.

Overall, Tiny Home developments have historically encountered NIMBYism. While the reasons for NIMBYism are multifaceted, accounting for NIMBYism is key to a project’s success [62]. Currently, there has not been direct opposition to the Edmonton Bridge Healing Program but it is advisable to remain proactive and be ready to implement effective public engagements, integration of local organizations, careful site selection, and successful monitoring in the hope of gaining public support [59,62]. These have previously been identified as effective strategies to combating NIMBYISM. Furthermore, the program could also follow in the footsteps of Occupy Madison and Social Bite Village, by using social media to improve its image and seek out celebrity endorsements [15,30].

### 6.5. Programs Addressing the Relationship Between Homelessness and Race?

Consideration of the intersections between race and homelessness has not been thoroughly examined in many cases of Tiny Homes. Of the cases examined, four of these studies do not have data on the percentage of non-white individuals in their program (Homes for Heroes, Social Bite, Kenton Women’s village, The Dwellings), nor do they outline programs that address any plan to create specific resources for homeless individuals of a systematically oppressed racial background. Dignity Village denotes that of non-white individuals present in their program, 1% are Black, and 3% are Indigenous [14]. This makeup reflects the homeless population distribution of Portland (where white individuals make up 79%, Black individuals 7%, and Indigenous make up 1%) but makes no mention of how these different groups are supported in terms of race [14]. Occupy Madison recognizes that “homelessness disproportionately impact[s] individuals of colour,” stating that of their population, 80% of homeless identify as non-white (74% of families and 48% of single males self-identifying as African American) [15]. However, “Occupy Madison’s board of directors resident community and surrounding neighbours are almost entirely white”, and did not include plans to address racial disparity [15]. In the case of Occupy Madison, they recognize that their program would possibly look different in terms of how they supported individuals had they included more diverse perspectives on their board of directors and therefore brought forth plans to introduce programs that addressed the intersection of homelessness and race.

When considering Edmonton’s makeup of homeless individuals, Indigenous people are vastly overrepresented, making up 48% of the total population [23,63]. This largely contrasts with the 5% of the total percentage of Indigenous people within Edmonton [23,63]. This pattern is seen in homeless patients in the ED. In 2016, it was found that 44% of homeless individuals seen in the ED are Indigenous [64]. While race is not an exclusionary factor of who can access Tiny Homes in our six case studies reviewed, nor is it in the proposed Bridge Healing Project, there has been evidence of race being a factor in housing discrimination [65]. As such, failing to consider how race impacts outcome in the success of homeless individuals in traditional housing models ignores the substantial impact that colonialism and systemic racism have had on general housing availability and the circumstances that result in homelessness [66,67,68]. To maximize the success of homeless individuals in such programs requires considerations of race [68].

In our evaluation of the proposed Bridge Healing Project, it does not include sufficient programming that specifically addresses the specific needs that Indigenous or other Non-White homeless individuals require. This represents a major flaw that needs to be addressed in the future.

### 6.6. Public Health

In addition to combatting general homelessness, what is unique about the Bridge Healing Program is its potential to have a positive impact on public health by reducing repeat visits to the ED, decreasing length of stays, and reducing hospital costs. The data on the correlation between deteriorating health and homelessness has become increasingly clear over the years. A report studying 1165 homeless Torontonians found that homeless residents substantially use hospital services at a higher rate compared to control groups (general population) with an average 9.1 ambulance encounters, and 2.0 ED visits per homeless person per year [69]. These ED visits appear to be caused by a small subset of the homeless population who are regularly returning to the ED. There are multiple reasons why patients experiencing homelessness visit the ED more, including lacking a family doctor, chronic health conditions, low socioeconomic standing etc. [70,71] Similarly, a study conducted in New York indicated that homeless patients also stayed an average of 36% longer than control patients [72]. Reasons reported included not having a place to send homeless patients post-discharge and physicians’ desire to keep homeless patients for follow-up care, knowing that they are less likely to return if they leave.

These problems are a major contributor to increased healthcare costs [72,73]. In fact, it is estimated that homeless people cost the ED $13,000 to $42,000 (CAD) per person per year [74]. It has been predicted that if British Columbia alone provided adequate housing and support services to all their homeless population, they would save ~$211,000,000 (CAD) annually [75]. By providing patients with a home and support services, this helps fulfill the concerns of physicians and also fulfills the social determinants (i.e., economic stability, housing, education, food, community, and health care) of health which are vital to improving health [76]. Consequently, patients will be less likely to return to the hospital or stay [76]. This would then mitigate both healthcare costs and ED overcrowding. Further evidence of this is shown by how, after the University of Illinois Hospital partnered with a housing center in 2015, they saw a reduction in ED visits by a 41% and a 46% cost reduction [77]. From 2017 to 2019, over 900 US hospitals have since followed suit and have begun investing in similar programs [77]. In short, the Bridge Healing Program represents a cost-efficient method that will improve the public health capacity in the community.

### 6.7. Evaluation of the Program

As of August 2020, there are no formal plans to determine how the Edmonton Bridge Healing Program will be evaluated but it has been proposed among team workers that one of the Capstone teams will be responsible. This team hopes to conduct updates and surveys (similar to KWV and Dignity Village) wherein they track how many people use the Program, the average length of time, and costs, and the team will also interview residents to determine user satisfaction [15,37]. Another potential idea is the use of their app, which will provide users with opportunities to give feedback and recommendations.

Through our research, we have identified some key parameters that have been shown to be good indicators of an affordable housing program’s relative success [78,79,80]. These are shown in Table 2.

### 6.8. The Ten Year Plan to End Homelessness and the Context of Edmonton

First proposed in 2009, “The Ten Year Plan to End Homelessness” was an Edmonton initiative intended to eradicate homelessness in the city by 2019 [81]. Based on the “Housing First” philosophy, the Edmonton Ten Year Plan was intended for people to move into long term housing as quickly as possible, while simultaneously providing services that would promote independence [82]. Their goals involved building 1650 affordable housing units by 2019, reducing the need for emergency shelters by 50%, and giving all people living on the street the option of permanent/supportive housing by 2011 [82]. Within those 10 years, the project saw minor successes, which included housing over 8400 people and the construction of 1263 private housing units [82]. Despite this, the project was unable to achieve its lofty ambitions as 1600 Edmontonians still remain homeless, they were unable to provide 387 promised units, and the number of people staying at homeless shelters had increased to 2006 levels.

One of the major reasons for this failure was the lack of funding that the program received, which consequently led to a shortage of affordable homes [83]. The initial proposal required an upfront investment of $402 million and operating costs of $568 million, but only ~50% of these funds were ultimately provided by the city [82]. In an attempt to realize these goals, an updated Ten Year Plan was proposed in 2018 which called for $230 million capital costs and $300 million operation funds. Listening to public opinion and learning from their lessons, the new project hopes to reduce the amount of funding necessary by focusing more on building affordable housing units and decreasing the reliance on emergency shelters, as their research found low intensity projects were most beneficial and cost-efficient (other cost-saving methods have been proposed as well). In order to end chronic and episodic homelessness, their targets include providing all “rough sleepers” with outreach and appropriate housing/support by 2018, connecting those entering homeless serving systems to housing within 21 days, and housing 4000 people by 2020 [82].

With regards to the Edmonton Bridge Healing Program, we see that this new proposal aligns with the goals of the city. Both subscribe heavily to a housing-first initiative and the Edmonton Bridge Healing Program will serve as a means of outreach to obtain affordable homes. Furthermore, when the city conducted a survey of 1600 community members, they found that increased affordable housing units with supports (namely addiction and mental health supports) were one of their highest priorities. As the Edmonton Bridge Healing Program intends to provide support to all graduates even after graduates begin independently living, it appears that this new program also meets the demands of the public.

## 7. Conclusions

After reviewing the case studies of the six Tiny Villages, their experiences point to a net benefit for homeless people in Canada. The villages are all unique and adapted to fit the specific needs and circumstances of the local environments and policies.

From the information we gathered, we built an initial (ideal) model for those seeking to design a Tiny Village. We conclude that an ideal model should have the following elements:

1. A Collaborative community wherein residents can work with staff to better the village as a whole. Case studies have shown that this fosters a sense of equity among community members and can be a defining feature of the project.
2. Public support or at the very minimum, strategies that improve public awareness and public relations on the benefit of Tiny Villages for the homeless.
3. Adequate funding for all housing and services costs.
4. The availability of affordable permanent or semi-permanent housing for residents upon graduation from Tiny Villages programs.

Applying these lessons to the Edmonton Bridge Healing Program, we conclude that the project has competently collaborated with local organizations to provide temporary housing, occupational opportunities, and health services to the homeless. Efforts to secure funding to maintain and expand the program are sought through outreach to investors, social and capital marketing and eventually through support from health care organizations and the public health system.

The program also anticipates relatively short stay times for residents compared to examples in the case studies, but this might be offset by the increasing amount of time spent by the program in helping find affordable housing for their residents. While this project has not encountered community opposition yet, the project should start making attempts to educate the public more about the initiative and be prepared to address potential hostilities as the project moves forward. One limitation of the Bridge Healing program is their current lack of consideration regarding ethnicities and race, despite Indigenous homelessness being greatly over-represented in Edmonton. Although there is a lack of data on how race/culture play in the Tiny Home projects we looked at, race/culture are important factors that affect inclusion and success of individuals in more traditional housing models/projects. In turn, the Bridge Healing Program should acknowledge and be aware that race/culture may have an impact on the project going forward. Even considering these limitations, the successful deployment of Tiny Villages to temporarily house homeless residents has the potential to effectively reduce the number of repeat ED visits from homeless residents seeking shelter and, consequently, to help diminish healthcare costs and alleviate ED overcrowding while also helping to combat homelessness in Canada.

## Figures and Tables

**Table 1:** Characteristics of some Tiny Villages across the world.

Name of Village	Price Per Month ($)	Size	Source of Funding	Stay Time	Services
Dignity Village	$35 and 10 h volunteer work per week	48 to 96 sq. feet units [9]	Independent	≤2 years	Limited
Occupy Madison	32 h initial volunteer work followed by 10 h per week	98 sq. feet	Independent	Permanent Housing	Limited
Homes for Heroes	n/a	Less than 300 sq. feet	ATCO, Mustard Seed, Veterans Affair	≤2 years	Yes
Social Bite Village	n/a	n/a	Social Bite Fund (charity), sandwich shop, corporate sponsorship	12–18 months	Yes
Kenton Women’s Village	n/a	32 sq. feet	Catholic Charities (CC)	Variable	Yes
The Dwellings	30% income	220–410 sq. feet	Non-profit	Variable	Yes

**Table 2:** Parameters to Include in the Edmonton Bridge Healing Program Evaluation.

Global Wellness Factors	Use of External Programs	Self-Sufficiency
General health Mental health and psychological functioning Substance use	Use of health services (emergency services, hospital use) Involvement with justice services (police, criminal justice, etc.) Food bank	Level of resident self-sufficiency (clients’ perspective) Level of resident self-sufficiency (staffs’ perspective)
